# Factors Associated with Help-seeking Behavior among U.S. Chinese Older Adults who Experienced Elder Mistreatment

**DOI:** 10.1007/s10903-025-01716-8

**Published:** 2025-07-07

**Authors:** Ying-Yu Chao, Jin Young Seo, Peijia Zha, Yu-Ping Chang

**Affiliations:** 1 School of Nursing, Rutgers, the State University of New Jersey, 180 University Avenue, Newark, NJ 07102-1803 USA; 2 Rutgers Institute for Health, Health Care Policy and Aging Research, 112 Paterson St, New Brunswick, NJ 08901 USA; 3 Hunter-Bellevue School of Nursing, Hunter College, CUNY 425 E. 25th St, New York, NY 10010-2590 USA; 4 School of Nursing, The State University of New York, University at Buffalo, 3435 Main St. Wende Hall, Buffalo, NY 14214-8013 USA

**Keywords:** Elder mistreatment, Older adults, Chinese Americans, Immigrants, Help-seeking

## Abstract

Elder mistreatment (EM) is an important public health issue. Guided by the Andersen’s Behavioral Model of Health Services Use, this study aimed to examine factors that impede or facilitate help-seeking behaviors among U.S. Chinese older adults who reported EM. Data were from the Population Study of Chinese Elderly in Chicago (PINE). Different types of mistreatments and help-seeking behaviors were assessed. Descriptive statistics and multinominal logistic regression were used to examine the factors associated with help-seeking. Among 450 participants who reported EM, 46.55% experienced psychological mistreatment, 29.91% had financial exploitation, 14.92% had caregiver neglect, 4.68% had physical mistreatment, and 1.11% had sexual mistreatment. Compared to those who did not seek help, EM victims who were female, married, perceived more social support, experienced financial exploitation, and experienced caregiver neglect were more likely to seek help from informal resources. EM victims who had higher income and perceived more social support were more likely to seek formal resources. EM victims who were female, experienced financial exploitation, and experienced caregiver neglect were more likely to seek help from both resources. Our findings reported the predisposing factors, enabling resources, and need factors that impacted seeking help from informal or/and formal resources among mistreated older Chinese Americans. Immigrants may be exceptionally socially isolated, and culturally specific barriers may substantially impact their motivations to seek help. Our findings suggested that greater attention to the provision of informal and formal social support can increase the effectiveness of prevention and intervention strategies in this vulnerable population.

## Background

Elder mistreatment (EM) is a single, repeated action, or lack of appropriate action within any relationship that causes harm or risk of harm to an older adult [1]. Common types of mistreatment are physical mistreatment (e.g., hitting, pushing, slapping, etc.), psychological/emotional mistreatment (e.g., yelling, threatening, etc.), financial exploitation (e.g., misuse, mismanagement or exploitation of property, belongings, or assets, etc.), sexual mistreatment (e.g., unwanted touching, etc.), and neglect (e.g., ignoring older adult’s needs, etc.) [2].

Risk factors for EM have been documented in the literature, though findings on sociodemographic predictors remain mixed. In the general U.S. older population, factors such as younger age, unmarried status, and low household income have been associated with increased risk of EM [3]. Studies also suggest disparities in EM prevalence across racial and ethnic minorities. For example, a population-based survey found that financial exploitation and psychological mistreatment were significantly more prevalent among African Americans than non-African Americans [4]. Similarly, the National Elder Mistreatment Study reported that non-White older adults were twice as likely as their White counterparts to report physical mistreatment, although no significant differences were observed for emotional or sexual mistreatment [5]. Among immigrant populations, certain cultural and social factors may further influence EM risk. A study of low-income Latino immigrants identified younger age, higher education, past abuse, and longer U.S. residency as significant risk factors [6]. More recently, emotional and financial mistreatment among older Korean Americans was found to be associated with younger age and lower levels of family solidarity [7]. Psychosocial risk factors of EM include aggressive behavior, social isolation, personality traits, self-neglect, and psychiatric illnesses (depression, alcohol abuse, past abuse). Health-related risk factors include functional/cognitive impairments, frailty, multimorbidity, and incontinence [8, 9]. The consequences of EM have been reported to be profound, ranging from various forms of physical and psychological symptoms and increased health-care consumption to premature mortality [10].

Help-seeking behavior refers to a victim admitting the threat to their identity and health problems, then searching for and requesting for help from informal sources (e.g., spouse, family, or friends) and formal services (e.g., law enforcement, legal/justice, or adult protective services) to cope with problems [11, 12]. It is estimated that 92.9–95.7% of EM episodes are unreported among older adults in the United States (U.S.). Most of older adult victims remain hidden from authorities and endure mistreatment [13].

Cultural beliefs and systemic barriers likely influence how EM is perceived and reported across different racial and ethnic groups. EM is often under-recognized and under-reported among Asian Americans [14]. Chinese Americans represent one of the largest Asian subgroups in the U.S. [15]. It is estimated that 88.6% of Chinese adults aged 55 and older were foreign-born, and about 30% immigrated to the U.S. after the age of 60 [15]. These demographic patterns raise important considerations regarding the role of cultural values and acculturation in shaping older immigrants’ perceptions of EM and their motivations to seek help. For instance, the unique Chinese cultural value of face-saving refers to an individual trying to maintain a positive self-image or reputation in interpersonal relationships [16]. Previous studies reported that Asian-American immigrants were more tolerant of abusive situations and less likely to seek help in order to sustain family harmony and family reputation compared to other racial/ethnic groups [17, 18]. Building upon the literature and recognizing the importance of incorporating cultural factor, the association between face-saving and help-seeking was suggested to be explored in the Chinese population [16].

The prevalence rate of EM varies from 13.9 to 25.8% among Chinese Americans [17]. Failure to identify the factors associated with help-seeking behaviors may delay opportunities to provide effective preventive and supportive strategies for this vulnerable population. To the best of our knowledge, the influence of predisposing characteristics, enabling resources, and need factors on help-seeking behaviors has not been examined among Chinese EM victims in the U.S. This proposed study aims to conduct secondary data analyses of the representative data of the Population Study of ChINese Elderly (PINE) to identify factors that impede or facilitate help-seeking of Chinese older adults who experienced EM in the U.S.

## Theoretical Framework

Andersen’s Behavioral Model of Health Services Use (BMHSU) [19] has been adapted and utilized extensively in many studies investigating the use of health services among diverse racial and ethnic populations [20, 21]. Andersen’s BMHSU model have been used to understand service use among older adults who experienced mistreatment [11]. This model proposes that the use of health services is predicted by a person’s predisposing characteristics, enabling resources, and level of need. Predisposing characteristics include individuals’ experiences and circumstances and determine their predisposition to illness and disease, such as demographic and social composition of communities. Enabling resources include personal, social, and community resources that facilitate or impede service utilization, such as social support, social network, and insurance coverage. Need factors include the nature and magnitude of the presenting problem or clinical need, such as comorbidities and self-rated health condition [19]. Cultural influence makes the detection of EM more complex among immigrants. Social stigma and the desire to save face often prevent Chinese EM victims from seeking help [22]. On the other hand, the acculturation process may change U.S. Chinese older adults’ endorsement of traditional Chinese cultural values. Given that cultural factors may play an important role in help-seeking, we tailored the BMHSU by adding the cultural factor domain.

## Methods

### Participants

The PINE study is a community-engaged, population-based epidemiological study which implemented extensive recruitment strategies that were culturally and linguistically appropriate through a community-based participatory research approach. Participants were self-identified Chinese older adults aged 60 years old and above who resided in the Greater Chicago area. Further details on the study methodology are well-documented [23]. The current paper analyzed the data from the second-wave PINE study that was collected from 2013 to 2015. Among the 3,132 older adult participants, EM was screened using a 10-item self-report instrument derived from the Whale–Sengstok Elder Abuse Screening Test (H-S/EAST) [24] and the Vulnerability to Abuse Screening Scale (VASS) [25]. Of these, 450 (14.37%) participants who reported experiencing any form of EM were included in the present study.

### Measures

#### Dependent Variable

An adapted General Help-Seeking Questionnaire (GHSQ) [26] and the Actual Help Seeking Questionnaire (AHSQ) [27] were used to measure EM help-seeking behaviors. The questions of sources of help include seven informal sources (e.g., partner, parent, children, family members, and friends) and ten formal sources (e.g., western primary care physicians, Chinese medicine doctors, mental health professionals, nurses, adult protective services, legal criminal justice system…, etc.). Participants were asked to whom they had gone for advice or help. Responses of help-seeking behaviors were categorized as: “informal sources of help”, “formal sources of help”, “both sources of help”, or ‘not seeking help”. The category of “both sources of help” indicated participants who sought help from both informal and formal sources.

#### Independent Variables

##### Predisposing Characteristics

Predisposing variables included victims’ age (in years), gender (male/female), marital status (unmarried/married), education (in years), and annual income (US $1,000).

##### Cultural Factors

Cultural Factors included acculturation (continuous) and face-saving (continuous). Acculturation was measured by a 12-item scale adapted from the Short Acculturation Scale for Hispanics, which higher scores indicated higher levels of acculturation (score range: 12 to 48). Participants had a low level of acculturation in the PINE study (Cronbach’s alpha = 0.88) [17]. Face-saving was measured with six items, adapted from [28], on a five-point Likert scale (alpha = 0.82). Total score ranges from 0 to 30, with a higher score indicating a greater level of face-saving. Participants had a high level of face-saving in the PINE study [16].

##### Enabling Resources

Enabling factors included health insurance (no/yes), perceived social support (continuous) and social networks. Perceived social support was measured using a 12-item social support scale drawn from the National Social Life, Health, and Aging Project (NSHAP), with higher scores reflecting greater social support [29]. The quantity of social networks was measured by social network size (continuous) and volume of contact (continuous). The quality of social networks was measured by emotional closeness (continuous). For social network size, participants were asked to list up to five network members with whom they discuss important matters. Volume of contact was the average contact frequencies that a participant talked to network members in the past year, ranging from 1 = less than once a year to 8 = every day. For emotional closeness, participants were asked, “How close do you feel is your relationship to this person?”, with ordinal responses ranging from 1 = not very close to 4 = extremely close [30].

##### Need Factors

Need factors included depression (Patient Health Questionnaire [31]; continuous), Anxiety (Hospital Anxiety and Depression Scale-Anxiety [HADS-A] [32]; continuous), and cognition (Mini-Mental State Examination [MMSE] [33]; continuous). Overall health status (continuous) which was measured by 5-item questions (e.g., general health, change health, quality of life) with higher score indicates poor overall health status. EM subtypes were measured using validated instruments. Psychological mistreatment (e.g., screamed or threatened participants) and physical mistreatment (e.g., hit, slapped, pushed, or kicked participants) were assessed using items from the modified Conflict Tactic Scale (CTS) [34]. Sexual mistreatment was measured with a single item asking participants if they have been touched in private areas when they did not want to be. Financial exploitation was assessed with 17 questions (e.g., if participants were forced/pressured to change/sign legal or financial documents) [35]. Caregiver neglect was assessed based on unmet needs in activities of daily living (ADL) and instrumental activities of daily living (IADL) [36, 37]. Each EM subtypes was treated as a categorical variable (no/yes). Multi-victimization was defined as exposure to two or more EM subtypes (no/yes).

### Analysis

Descriptive statistics were used to summarize the socio-demographic information and sources of help-seeking behaviors of the participants. Multinomial logistic regressions were conducted to examine the factors associated with EM help-seeking behaviors (informal sources of help, formal sources of help, and both sources of help). Not seeking help was treated as the reference group. Odds ratios (ORs) and 95% confidence intervals (CIs) were calculated. A significant level of *p* ≤.05 was used in analyses. All statistical analyses were conducted using SAS, Version 9.2 (SAS Institute Inc., Cary, North Carolina).

## Results

### Sample Characteristics

Among the victims, 209 (46.55%) experienced psychological mistreatment, 21 (4.68%) had physical mistreatment, five (1.11%) had sexual mistreatment, 134 (29.91%) had financial exploitation, and 67 (14.92%) had caregiver neglect. The mean age of these EM victims was 72.73 (SD 8.03, range 60 to 97). Of the victims, 56% were female and 72.38% were currently married. The mean years of education was 10.63 (SD 5.19), and the average income was US$2,080. 384 (85.33%) of victims had health insurance. Victims had a mean of 3.52 (SD 5.15) of PHQ-9, a mean of 3.82(SD 4.48) of HADS-A and a mean of 25.54 (SD 4.6) of MMSE.

192 (53.48%) of victims sought help from informal sources, 114 (31.75%) did not seek help from any sources, 41 (11.42%) sought help from both informal and formal sources, and 12 (3.34%) sought help from formal sources. The most common informal sources were adult children, followed by a partner and friends/neighbors. The most common formal resources were local community service centers, followed by the legal criminal justice system (Table 1).

**Table 1:** Characteristics of the PINE study participants reporting elder mistreatment (*N* = 450)

	Mean (SD)	*n* (%)
Predisposing Characteristics		
Age (years)	72.73 (8.03)	
Gender		
Male		198 (44)
Female		252 (56)
Marital status		
Unmarried		124 (27.62)
Married		325 (72.38)
Education	10.63 (5.19)	
Income (US $1,000)	2.08 (1.29)	
**Cultural Factors**		
Acculturation (score range: 12–48)	16.58 (5.52)	
Face-saving (score range: 0–30)	15.57 (4.44)	
**Enabling Resources**		
Health insurance		
No		66 (14.67)
Yes		384 (85.33)
Perceived social support (12–36)	28.52 (3.36)	
Social network size (0–5)	3.29 (1.31)	
Volume of contact (1–8)	6.62 (1.10)	
Emotional closeness (1–4)	3.24 (0.67)	
**Need Factors**		
Overall health status (1–5)	3.01 (0.83)	
Depression (PHQ-9: 0–27)	3.52 (5.15)	
Anxiety (HADS-A: 0–21)	3.82 (4.48)	
Cognition (MMSE: 0–30)	25.54 (4.60)	
Psychological mistreatment		
No		240 (53.45)
Yes		209 (46.55)
Physical mistreatment		
No		428 (95.32)
Yes		21 (4.68)
Sexual mistreatment		
No		444 (98.89)
Yes		5 (1.11)
Financial exploitation		
No		314 (70.09)
Yes		134 (29.91)
Caregiver neglect		
No		382 (85.08)
Yes		67 (14.92)
Help-seeking behaviors		359
Informal sources of help		192 (53.48%)
Formal sources of help		12 (3.34%)
Both sources of help		41 (11.42%)
Not seeking help		114 (31.75%)

SD = Standard deviation

### Help-seeking from Informal Sources

Compared to not seeking help, EM victims who were female (odds ratio [OR] = 3.73, 95% CI: 1.91–7.27, *p* <.001), married (OR = 2.25, 95% CI: 1.05–4.79, *p* <.05), perceived more social support (OR = 1.13, 95% CI: 1.03–1.25, *p* <.05), experienced financial exploitation (OR = 2.48, 95% CI: 1.09–5.63, *p* <.05), and experienced caregiver neglect (OR = 3.39, 95% CI: 1.12–10.25, *p* <.05) were more likely to seek help from informal sources. Conversely, EM victims who had larger social network sizes were less likely to seek help from informal sources (OR = 0.71, 95% CI: 0.56–9.91, *p* <.01) (Table 2).

**Table 2:** Multinomial logistic regression models predicting elder mistreatment help-seeking behaviors (*N* = 450)

	*Help-seeking Behaviors*		
	Informal sources	Formal sources	Both sources
	OR (95% CI)	OR (95% CI)	OR (95% CI)
**Predisposing Characteristics**			
Age (years)	0.99 (0.95–1.04)	1.12 (0.98–1.27)	1.03 (0.96–1.10)
Gender	3.73 (1.91–7.27)***	3.25 (0.49–21.74)	3.15 (1.12–8.89)*
Marital status	2.25 (1.05–4.79)*	0.72 (0.09–5.55)	1.19 (0.39–3.60)
Education	1.01 (0.95–1.08)	1.13 (0.92–1.40)	0.95 (0.86–1.05)
Income (US $1,000)	1.01 (0.80–1.27)	1.79 (1.07–3.00)*	1.19 (0.78–1.82)
**Cultural Factors**			
Acculturation	0.98 (0.93–1.04)	1.00 (0.87–1.16)	0.89 (0.80–1.00)
Face-saving	1.03 (0.97–1.10)	0.98 (0.80–1.20)	1.05 (0.96–1.16)
**Enabling Resources**			
Health insurance	1.01 (0.46–2.24)	0.15 (0.02–1.13)	1.33 (0.36–5.00)
Perceived social support	1.13 (1.03–1.25)*	1.63 (1.20–2.21)**	1.06 (0.90–1.25)
Social network size	0.71 (0.56–0.91)**	0.62 (0.32–1.22)	0.88 (0.60–1.28)
Volume of contact	1.03 (0.76–1.39)	0.69 (0.31–1.50)	1.16 (0.73–1.86)
Emotional closeness	1.32 (0.80–2.16)	0.49 (0.14–1.64)	0.62 (0.29–1.32)
**Need Factors**			
Overall health status	1.02 (0.73–1.42)	1.72 (0.67–4.44)	1.09 (0.62–1.90)
Depression	1.06 (0.99–1.14)	1.00 (0.81–1.25)	1.02 (0.92–1.14)
Anxiety	0.92 (0.85–1.01)	0.99 (0.80–1.24)	1.00 (0.88–1.14)
Cognition	0.99 (0.92–1.07)	1.01 (0.80–1.27)	1.07 (0.96–1.20)
Psychological abuse	1.47 (0.72–3.03)	0.05 (0–1.34)	1.34 (0.33–5.39)
Physical abuse	1.75 (0.41–7.44)	0.29 (0.01–7.15)	4.27 (0.66–27.78)
Sexual abuse	0.91 (0.07–12.18)	33.32 (0.39–2837.2)	0 (0 - I)
Financial exploitation	2.48 (1.09–5.63)*	0.76 (0.10–5.55)	11.02 (3.16–42.81)***
Caregiver neglect	3.39 (1.12–10.25)*	0.58 (0.03–10.36)	6.99 (1.43–34.06)*

Note. Not seeking help as reference group. OR = odds ratio; CI = confidence interval

*p <.05, **p <.01, ***p <.001

### Help-seeking from Formal Sources

Compared to not seeking help, EM victims who had higher incomes (OR = 1.79, 95% CI: 1.07-3.00, *p* <.05) and perceived more social support (OR = 1.63, 95% CI: 1.20–2.21, *p* <.01) were more likely to seek help from formal sources (Table 2).

### Help-seeking from both Sources

Compared to not seeking help, EM victims who were female (OR = 3.15, 95% CI: 1.12–8.89, *p* <.05), experienced financial exploitation (OR = 11.02, 95% CI: 3.16–42.81, *p* <.001), and experienced caregiver neglect (OR = 6.99, 95% CI: 1.43–43.06, *p* <.05) were more likely to seek help from both sources (Table 2).

## Discussion

### Sources of Help-seeking Behaviors

Similar to the previous review study [38–40], most victims favored contacting informal sources over formal sources. Only a small number of our sample sought help from medical, social, and legal professionals. The rate of help-seeking from formal sources in this study (3.34%) was lower than the rate (11.1%) reported in the U.S. National Elder mistreatment study [41]. About one-third of our sample did not seek help at all. Previous studies have reported reasons why immigrants who experienced EM are reluctant to seek help, including language and cultural barriers, inefficiency of support systems, undocumented immigrant status, lack of health insurance coverage, lack of knowledge about health care access, failure to recognize abusive experiences, social isolation, stigma associated with EM, lack of knowledge of resources, and insufficient financial resources [22, 40].

### Facilitators and Barriers of Help-seeking Behaviors

In this study, women were more likely to seek informal help compared to men. Some challenges for men to disclose abuse experience could be due to social factors (traditional gender roles and norms), personal factors (shame, identity impacts) and practical barriers (cost, insurance, and scheduling issues) [42]. In Chinese culture, men play masculine roles, making them less likely to share personal information and more inclined to have difficulty relinquishing control. These challenges were also a pivotal observation in a cohort study on the help-seeking behaviors of EM victims in a primary care setting [43]. Victims in our sample who were married were more likely to seek informal help. It is possible that family/partners are the victims’ primary sources of help. Family members usually provide victims with emotional support and information about available community resources [44]. This tendency may be especially pronounced among Chinese immigrants, given strong cultural values of familism and interdependence [45]. Many rely on family rather than formal services due to language barriers and limited familiarity with the U.S. elder mistreatment reporting system. Furthermore, older women may be particularly hesitant to report abuse if they are financially dependent on their spouses.

In accordance with the findings of a previous systematic review that income is associated with professional care and treatment in older adults [46], we also found that victims with higher household incomes were more likely to seek help from formal sources. As such, financial assessment and tailored follow-up are recommended for low-income household.

With regard to *cultural factors*, a previous study found that many Chinese older adults believe that reporting abuse and seeking help may negatively affect family relationships. As such, they were reluctant to report abuse in order to retain their family’s harmony and reputation [23]. Surprisingly, our findings did not support the influence of acculturation and face-saving on EM help-seeking behavior in this vulnerable population. Filial piety, another important cultural factor in Chinese culture, emphasizes that younger generation must respect and provide care for the older generation. However, perspectives on filial piety may change under the influence of Western culture. Future studies should consider exploring the influence of filial piety on EM help-seeking behavior in this population.

The facilitator in *enabling resources* of EM help-seeking behaviors is perceived social support. Immigrants often experience a significant loss in social support when they move to a new country. Many EM victims are initially unaware of the available social support services. They do not know where and how to seek formal services. Additionally, they may not use the available formal services or may have difficulty accessing these services due to age-related disabilities [47, 48]. Social support can promote resilience in immigrants by increasing their sense of belonging, decreasing exposure to and the effects of racism, and improving access to information and formal services [22, 49]. Evidence supports our findings that a sufficient social support system can effectively promote informal and formal help-seeking behaviors in EM victims [39]. However, social network size in our sample was found to be a barrier to EM help-seeking. While the PINE Study dataset does not explicitly specify the relationship between the perpetrator and victim (e.g., family vs. non-family), caregiving in this population is primarily provided by family members. In such cases, the social networks of perpetrators may overlap significantly with those of the victims, potentially exacerbating reluctance to seek help due to familial shame, obligation, or economic dependence. This pattern aligns with findings from Burnes et al. [11] which showed that larger perpetrator friendship networks deterred victim help-seeking. Victims may be reluctant to seek help because they have a high level of dependence on their perpetrator, making them fear losing support from the perpetrators’ social network.

*Need characteristics* were the strongest facilitators of EM help-seeking behaviors. Chinese older adults who experienced financial exploitation and caregiver neglect were more likely to seek help from informal sources. Victims who experienced financial exploitation found it easier to open up because financial issues are more publicly discussed and commonly experienced by others. They may feel less shameful seeking help [41]. Under the influence of filial piety in Chinese culture, conflicts between younger and older generations may be viewed as mistreatment by older immigrants [23]. In our sample, victims who felt upset about not being respected by their children may complain of caregiver neglect. They may receive empathy and support from the informal resources such as family and friends. However, immigrants may not be able to disclose abuse to formal services due to language barrier, insufficient financial resources, lack of awareness, cultural and religious factors, issues of cultural competence, and lack of diversity within frontline services [22, 40, 48]. A recent systematic review reported facilitators of victims’ formal help-seeking include provider knowledge, informal and formal support, accessibility (e.g., offering a helpline or the ability to text for help) to violence prevention agencies and organizations, and participants’ desire to provide protection and prevent future violence [39]. Nevertheless, it is important to increase screening for EM in clinical services and promote awareness of EM among U.S. Chinese older adults.

The findings of this study highlight the need for culturally sensitive interventions when addressing EM in diverse communities. In the Chinese American community, where values of familial interdependence and respect for older adults are deeply embedded, interventions should emphasize family-centered approaches that uphold dignity while addressing EM. For example, healthcare providers can collaborate with community leaders and trusted organizations to deliver culturally tailored workshops on EM awareness. Involving respected figures such as religious leaders or elder advocates may help reduce stigma around disclosure and reporting. Additionally, given the cultural tendency to view EM as a private family matter, outreach efforts should also focus on educating families about the signs of mistreatment and the availability of confidential support services. To facilitate help-seeking behavior, informal support systems such as community centers or social groups can be integrated to provide safe, non-threatening spaces for older adults to share their experiences. The use of multilingual resources and culturally competent healthcare professionals can further improve accessibility and trust. By aligning interventions with the cultural values of Chinese American community, healthcare providers can enhance engagement, reduce barriers to reporting, and foster long-term solutions to EM.

### Strengths and Limitations

This is the first study using the population-based epidemiological dataset to identify facilitators and barriers to EM help-seeking behaviors among U.S. Chinese older adults. The study examined the factors influencing both informal and formal help-seeking behaviors. In addition, the study builds on previous research [22, 23] to explore the role of cultural values in help-seeking behaviors among Chinese immigrants. There are some limitations to this study. First, this study only examined Chinese older adults living in the Greater Chicago area. Hence, the findings cannot be generalizable to those living in different geographic regions. Second, this study utilized a cross-sectional design. Future studies with longitudinal designs can improve the understanding of causal relationships between factors and help-seeking behaviors. Third, immigrants who arrive in a new country at different life stages may face different challenges and have different available resources. Future studies may compare factors affecting help-seeking behaviors by the length of immigration history.

### New Contribution To the Literature

In summary, our findings suggest that predisposing factors, enabling resources, and need factors impact the likelihood of seeking help from informal and/or formal resources among older Chinese Americans who reported mistreatment. Abused Chinese immigrants in the U.S. may be exceptionally isolated, and culturally specific barriers may substantially impact their motivations to seek help. Greater attention to providing informal and formal social support can promote the safety and well-being of older adults and increase the effectiveness of prevention and intervention strategies in this vulnerable population.
